# Defining the role of exertional hypoxemia and pulmonary vasoconstriction on lung function decline, morbidity, and mortality in patients with chronic obstructive lung disease – the PROSA study: rationale and study design

**DOI:** 10.1186/s12890-024-03074-x

**Published:** 2024-05-30

**Authors:** Rainer Böger, Juliane Hannemann

**Affiliations:** 1 Institute of Clinical Pharmacology and Toxicology, University Medical Center Hamburg-Eppendorf, Hamburg, Germany; 2 Institute DECIPHER, German-Chilean Institute for Research on Pulmonary Hypoxia and its Health Sequelae, Hamburg, Germany

**Keywords:** Chronic obstructive lung disease, Exercise testing, 6-minute walk test, Exertional desaturation, Endothelium, Outcome, Prospective study

## Abstract

**Background:**

Chronic obstructive lung disease (COPD) has diverse molecular pathomechanisms and clinical courses which, however, are not fully mirrored by current therapy. Intermittent hypoxemia is a driver of lung function decline and poor outcome, e.g., in patients with concomitant obstructive sleep apnea. Transient hypoxemia during physical exercise has been suggested to act in a similar manner. The PROSA study is designed to prospectively assess whether the clinical course of COPD patients with or without exertional desaturation differs, and to address potential pathophysiological mechanisms and biomarkers.

**Methods:**

148 COPD patients (GOLD stage 2–3, groups B or C) will undergo exercise testing with continuous pulse oximetry. They will be followed for 36 months by spirometry, echocardiography, endothelial function testing, and biomarker analyses. Exercise testing will be performed by comparing the 6-min walk test (6MWT), bicycle ergometry, and a 15-sec breath-hold test. Exertional desaturation will be defined as SpO_2_ < 90% or delta-SpO_2_ ≥ 4% during the 6MWT. The primary endpoint will be the rate of decline of FEV1(LLN) between COPD patients with and without exertional desaturation.

**Discussion:**

The PROSA Study is an investigator-initiated prospective study that was designed to prove or dismiss the hypothesis that COPD patients with exertional desaturation have a significantly more rapid rate of decline of lung function as compared to non-desaturators. A 20% difference in the primary endpoint was considered clinically significant; it can be detected with a power of 90%. If the primary endpoint will be met, exercise testing with continuous pulse oximetry can be used as a ubiquitously available, easy screening tool to prospectively assess the risk of rapid lung function decline in COPD patients at an early disease stage. This will allow to introduce personalized, risk-adapted therapy to improve COPD outcome in the long run. PROSA is exclusively funded by public funds provided by the European Research Council through an ERC Advanced Grant. Patient recruitment is ongoing; the PROSA results are expected to be available in 2028.

**Trial registration:**

The PROSA Study has been prospectively registered at clinicaltrials.gov (register no. NCT06265623, dated 09.02.2024).

## Background

Chronic obstructive pulmonary disease (COPD) is among the most prevalent chronic pulmonary diseases in the European population. Data from six European countries within the global Burden of Lung Diseases (BOLD) study suggest that COPD prevalence varies between 18 and 28% in the adult population aged 40 years and above [1]. The global number of COPD cases was estimated as 384 million in 2010, with a worldwide prevalence of 11.7% (95% CI 8.4–15.0%) and some three million deaths annually [2, 3]. The WHO recently projected the annual death rate attributable to COPD and related conditions to rise to as many as some 5 million for 2060 [4].

**Fig. 1:**
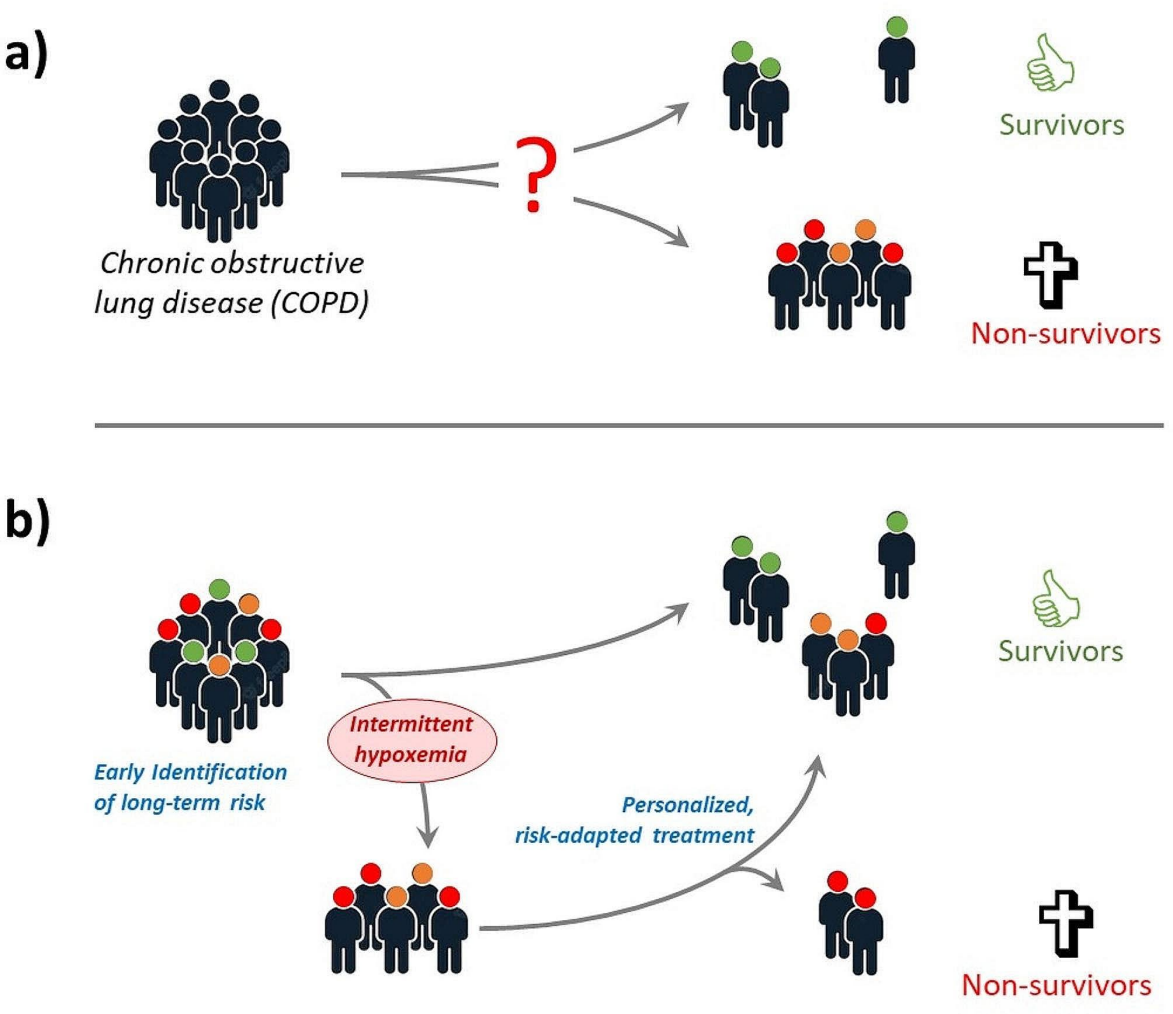
Currently all COPD patients are being treated by the same drugs irrespectively of their individual risk of rapid or slow progression of lung function decline, because we lack prognostic diagnostic tests (**a**). Thus, some patients have a rapid progression of their lung disease and a high risk of early mortality, but we can find out who these individuals are only when clinical events set in, depriving us of the opportunity of early, personalized and risk-adapted treatment that might bear chances to improve patients’ prognosis. Identification of ling-term risk in an early disease stage (**b**) will allow to allocate personalized, risk-adapted treatment to those with a high risk and offer the perspective of reducing the mortality rate of COPD.

To counteract this predicted increase in COPD prevalence and mortality, there is an urgent need to identify COPD patients with a high risk of rapid disease progression at an early disease stage. To this end, we need to even better understand the pathophysiology of the disease; it will help us to identify novel targets for pharmacological therapy. Figure 1 schematically depicts the historical approach to COPD treatment and a possible future strategy for improved risk-adapted, personalized care of COPD patients.

Recent research has shown that the genesis of COPD starts early in life, resulting in varying lung function trajectories that may or may not ultimately lead to COPD in later life [5]. A steeper annual decline in FEV1 is associated with a higher risk of chronic respiratory disease and total mortality [6].

Traditional understanding has been that hypoxemia and pulmonary arterial hypertension may not develop but late in the disease course of COPD; however, the 2022 revision of the Global Initiative for Chronic Obstructive Lung Disease (GOLD) Guideline acknowledged that “[…] perturbations in pulmonary vasculature are major, but under recognized, drivers of symptoms and exacerbations in COPD” [7]. Indeed, vascular dysfunction and impaired endothelial nitric oxide (NO) production have been shown to occur early during the disease course [8].

The well-described poor prognosis of patients with overlap syndrome (i.e., the simultaneous presence of COPD and obstructive sleep apnoea syndrome [OSAS]) and the finding that patients with overlap syndrome show a faster decline of lung function (FEV1/FVC) over time than COPD patients without OSAS strongly underscore the hypothesis that intermittent hypoxemia is a major pathogenic factor in COPD development and progression [9, 10]. Further, both COPD and OSAS are associated with major cardiovascular co-morbidity which – importantly – is already detectable at early disease stages [8, 11] and is aggravated when both COPD and OSAS are present [10]. Hypoxemia, even when occurring intermittently, causes dysfunctional endothelium-dependent, NO-mediated vascular regulation in the pulmonary and systemic circulation in both diseases [12, 13]. Transient hypoxemia evokes adaptations in the pulmonary and systemic micro- and macrocirculation [14], and it changes pulmonary gene expression in a manner mimicking chronic lung diseases [15]. Therefore, intermittent hypoxemia may drive COPD progression via acting on the pulmonary endothelium, which has been named the “orchestra conductor in respiratory diseases” [16]. By this way, the pathophysiological mechanisms set off by hypoxia in the lungs are relevant drivers of pulmonary hypertension and right ventricular hypertrophy and failure on the one hand, but they also promote systemic cardiovascular disease and mortality.

Exertional hypoxemia as a model for provoking intermittent hypoxemia has been tested in several small studies using diverse exercise approaches. Traditionally, the 6-minute walk test (6MWT) has been used to test exercise capacity and exertional hypoxaemia in COPD patients [17]. Exertional desaturation during the 6MWT has been associated with rapid lung function decline [18] and high mortality rate [19]. However, a 15-second breath-hold-test (15-sec BHT) has also been proposed as an easily feasible alternative to test COPD patients for the presence of exercise-induced hypoxaemia [20]. Finally, bicycle ergometry remains the gold standard for exercise testing in clinical medicine; however, performing this test requires more sophisticated equipment and trained personnel than the 6MWT or the 15-sec BHT. Exertional hypoxaemia during bicycle ergometry has been shown to reflect pulmonary artery pressure in COPD patients [21].

The PROSA study (Prospective observational study to assess the influence of intermittent hypoxaemia on lung function decline, morbidity, and mortality in COPD patients) was designed to generate definite evidence for the association of exertional hypoxaemia with lung function decline and outcome in COPD patients. Beyond this, the study aims to elucidate pathophysiological mechanisms underlying this relationship and potential biomarkers that may help to better identify COPD patients at high risk of rapid disease progression at an early time point during their disease course.

## Methods

### Study design

PROSA is a monocenter, observational, prospective cohort study. 148 patients with chronic obstructive lung disease (COPD) stages 2–3, groups B and E according to GOLD [7] will be enrolled in this clinical study. We expect that one third of our study cohort will experience exertional hypoxemia based on incidence rates reported in previous studies [22, 23]. Patients will be recalled to the study site once per year for a total of 36 months of follow-up.

The primary endpoint of the study will be the difference in FEV_1_ (LLN) over time in COPD patients with exertional desaturation versus those without exertional desaturation (exertional desaturation will be defined as oxyhemoglobin saturation by pulse oximetry (SpO_2_) < 90% or a drop of SpO_2_ ≥ 4% during a six-minute walk test [19].

Secondary endpoints have been defined as follows: (1) The difference in all-cause mortality over time in COPD patients with exertional desaturation versus those without exertional desaturation. (2) The difference in COPD-related mortality over time in COPD patients with exertional desaturation versus those without exertional desaturation. (3) The difference in frequency of hospital admissions for exacerbation of COPD in COPD patients with exertional desaturation versus those without exertional desaturation. (4) The difference in frequency of hospital admissions for causes other than exacerbation of COPD in COPD patients with exertional desaturation versus those without exertional desaturation. (5) The sensitivity and specificity of plasma biomarkers at baseline to predict the prevalence of exertional desaturation in COPD patients. (6) The sensitivity and specificity of plasma biomarkers at baseline to prospectively predict the slope of lung function decline in COPD patients. (7) The sensitivity and specificity of plasma biomarkers at baseline to prospectively predict the mortality rate in COPD patients. (8) The difference in incidence and extent of exercise hypoxemia between carriers and non-carriers of single nucleotide polymorphisms in genes of the L-arginine – dimethylarginine pathway. (9) The difference in positive and negative predictive value of bicycle ergometry, 6-minute walk test, and 15-sec breath-hold test to discriminate between COPD patients with exertional desaturation versus those without exertional desaturation.

### Determination of endpoints

#### Spirometric assessment of lung function

Lung function will be assessed by spirometry according to pneumological routine protocols. FEV_1_ and FVC will be recorded amongst other routine spirometric parameters and interpreted according to the latest edition of joint European Respiratory Society / American Thoracic Society Guideline on spirometry [24, 25] as the best values reached by a patient during three repeated spirometry runs on each clinical investigation to assess pulmonary function. FEV_1_ results will be analyzed against age- and sex-controlled normal ranges (LLN = lower limit of the normal range) [26], and their change between baseline investigation and the last investigation during follow-up of this study will be calculated to assess the percent change in FEV_1_ (percent of LLN), which is the primary end-point of the study.

#### Assessment of exertional hypoxemia

Three different approaches will be used to assess if patients experience intermittent hypoxemia. All of these clinical approaches have been used before in clinical investigations with COPD patients, but the diagnostic utility of these different procedures has not been assessed in direct comparison. First, patients will be asked to perform a 15-second breath-holding test under supervision by study personnel while arterial oxygen saturation is continuously recorded with a pulse oximetry device clipped to a fingertip [20]. After a few minutes of rest, patients will perform a 6-minute walk test according to the guidelines of the American Thoracic Society [27] under supervision by study personnel while arterial oxygen saturation is continuously recorded with a pulse oximetry device clipped to a fingertip. The walk test will be performed indoor, and the total distance walked during 6 min by the patient will be recorded together with the lowest arterial oxygen saturation during the test. After another resting period, patients will undergo bicycle ergometry according to a protocol published by Miki and colleagues [21]. Ergometry will be performed under supervision by an investigator while arterial oxygen saturation is continuously recorded with a pulse oximetry device clipped to a fingertip. The exercise protocol will consist of a steady-state resting period of at least 3 min, followed by workload increments of 5 W every minute starting at 0 W until patient exhaustion in the sitting position using a bicycle ergometer (Ergoselect 100P, Ergoline, Bitz, Germany). Arterial oxygen saturation will be continuously recorded with a pulse oximetry device clipped to a fingertip; blood pressure readings will be done every minute during the last 15-s period of each stage.

#### Determination of NO-mediated vasodilation in the brachial artery

Flow-mediated vasodilation in the brachial artery is a non-invasive measure of endothelial nitric oxide release that has been shown to be an early sign of systemic cardiovascular disease [28]. This investigation will be performed according to the guidelines published by the International Brachial Artery Reactivity Task Force [29]. The brachial artery will be imaged transcutaneously with a high-resolution ultrasound probe on the right forearm (Samsung HV70 EVO; Samsung, Schwalbach, Germany). The mean brachial artery diameter will be measured on a 2 cm longitudinal segment with Brachial Analyzer 6 software (Medical Imaging Applications LLC, Coralville, Iowa, USA). The difference in brachial artery diameter from baseline to hyperemia after 5 min of suprasystolic occlusion at the upper arm with a blood pressure cuff will be calculated to express endothelium-dependent, NO-mediated vasodilation (%). After ten minutes of rest, another baseline image will be recorded, followed by the administration of one sublingual spray burst of glyceryl trinitrate, and image acquisition at 60–90 s. later to assess endothelium-independent, NO-mediated vasodilation (%).

#### Echocardiographic assessment of the right ventricle and pulmonary arterial pressure

Echocardiography will be performed according to routine cardiological imaging procedures using a Samsung HM70 EVO echocardiography system with a PN1-5 phased-array probe (1–5 MHz). Imaging studies will include measurements of cardiac output, right ventricular pressure gradient, and estimations of right atrial pressure.

#### Biomarkers and genotyping

Blood samples from an antecubital vein and capillary blood will be collected for analysis of L-arginine-related biomarkers, genotyping of genes in L-arginine metabolic pathways, platelet aggregation, and routine clinical chemical and hematological parameters.

### Ethical conduct

The PROSA study will be conducted in agreement with the principles set forth in the Declaration of Helsinki in its latest revision adopted in Fortaleza, 2013. The study protocol has been submitted for review to the responsible Ethics Committee (Ethics Committee of the Hamburg Board of Physicians) and received a positive vote (2024-101240-BO-ff; dated 21.02.2024). The protocol has been registered with clinicaltrials.gov (register no. NCT06265623, dated 09.02.2024).

### Study conduct and funding

The PROSA study is exclusively being funded by the European Commission as part of the European Research Council Advanced Grant Project “NO PRESSURE” (Regulation of the L-Arginine – ADMA – Nitric Oxide (**NO**) Pathway in the **P**ulmonary Vascular **Res**ponse to Hypoxia and its Role for **Sur**vival of High-Risk COPD Pati**e**nts; Proposal NO. 101,096,706).

Patients who are selected by their primary physician or pulmonology specialist as suitable for participation in the study and who are willing to undergo screening for the study will be screened by the investigators according to the inclusion and exclusion criteria. Patients who fulfil the inclusion and exclusion criteria will be informed about the scope, aims, and possible risks of the study, and after having given their written informed consent, will undergo baseline investigation (study day 1).

Investigations on study day 1 will comprise peripheral venous blood sampling and capillary blood sampling, assessment of endothelial function by measuring flow-mediated vasodilation on the forearm by ultrasound, echocardiography, spirometry, and exercise testing. The total duration of these studies is estimated to take 2.5–3 h per patient.

After completing clinical investigations on study day 1, patients will be released into ongoing primary medical care. The second investigation (study day 2) will be scheduled at 12 months after the first study day, and additional studies will take place at 24 months (study day 3) and 36 months (study day 4) after the first study day. On each of these follow-up study days, patients will be asked about their health status during the preceding 12 months, with special focus, but not exclusively, on clinical symptoms and possible exacerbations of their COPD. Thereafter, the same clinical investigations as described for study day 1 will be repeated in identical manner. After the end of study day 4, i.e. at 36 months, the study will be completed for each patient.

### Statistical analysis

Statistical analyses will be performed using the SPSS Version 27 software package (IBM Corporation, Armonk, NY, USA) and GraphPad Prism (version 6.01, GraphPad Software, San Diego, CA, USA) in the investigational center. All variables will be tested for normal distribution using the Kolmogorov–Smirnov test. Data will be expressed as mean with standard deviation (SD). Differences between groups will be tested for significance using the nonparametric Mann–Whitney U test for two groups or the Kruskal–Wallis analysis of variance for more than two groups. The Chi^2^ test will be used for comparison of categorical variables between groups. Logistic regression will be used to assess the relationship between exertional hypoxemia and mortality. The nil hypothesis for statistical evaluation of the primary endpoint will be that the difference in FEV_1_ (LLN) at 3 years of follow-up will not be different between COPD patients with exertional desaturation versus those without exertional desaturation. Exertional desaturation will be defined as oxyhemoglobin saturation by pulse oximetry (SpO_2_) < 90% or a drop of SpO_2_ ≥ 4% during a six-minute walk test [19].

Survival analyses will be performed using Kaplan–Meier curves comparing patients with or without exertional hypoxemia, and with ADMA and SDMA above or below the cut-off value determined in receiver-operated curve (ROC) analyses. Hazard ratios (HR) and 95% confidence intervals (CI) will be calculated by multivariable-adjusted logistic regression analyses. For all tests, *p* < 0.05 will be considered statistically significant. A correction of p will be performed for multiple comparisons when adequate.

### Sample size discussion

Sample size estimation was based on analysis with repeated-measures 2-way ANOVA (baseline – 1 year – 2 years – 3 years). We assume that one third of our study cohort will experience exertional hypoxemia as reported in previous studies [22, 23], and we estimate that a 20% difference in the primary endpoint will be of clinical significance. To prove or dismiss the primary endpoint with α = 0.05 and a power of 0.90, a total sample size of 148 COPD patients stages 2–3, groups B and E according to the current GOLD guideline [7] will be included in this study after accounting for an expected drop-out rate of up to 15%.

## Discussion

After decades with no major advances in the treatment of COPD beyond long-term bronchodilator therapy, better understanding of the pathophysiology of this disease has recently led to a more differentiated approach to COPD therapy. Innate lung function trajectories have been described, explaining some the variability of lung function findings in COPD patients and healthy individuals [5, 30]. A subgroup of COPD patients with a contribution of immune mechanisms to lung function impairment has been described; these patients are characterized by elevated eosinophil leukocyte counts and/or high IgE levels; combined treatment with an inhaled corticosteroid and long-acting bronchodilator has been advocated for this subgroup [31]. The pulmonary vasculature has been identified as another important pathophysiological factor [16, 32]. However, clinically measurable pulmonary hypertension is rare in early disease states and increasingly frequently found only in advanced stages of the disease [33]. Nonetheless, intermittent hypoxemia-induced pulmonary vascular dysfunction may occur in early stages of COPD, when patients still are well compensated at rest, e.g. when patients are exercising [34]. Indeed, exercise-induced desaturation of arterial blood oxygen has been repeatedly described using a variety of exercise protocols, and it has been associated with accelerated lung function decline and increased mortality [17–21]. Hypoxemia, like hypoxia, may cause pulmonary vasoconstriction by acting on the endothelium-mediated regulation of pulmonary vascular tone, and cause transient increases in pulmonary arterial pressure. Hypoxia has been shown to induce an inflammatory phenotype in the lung and systemically [35] and affects gene expression in the lung similar to the changes occurring in chronic lung diseases [15]; it may thus promote pulmonary inflammation, aggravate progression disease and stipulate systemic co-morbidities.

**Fig. 2:**
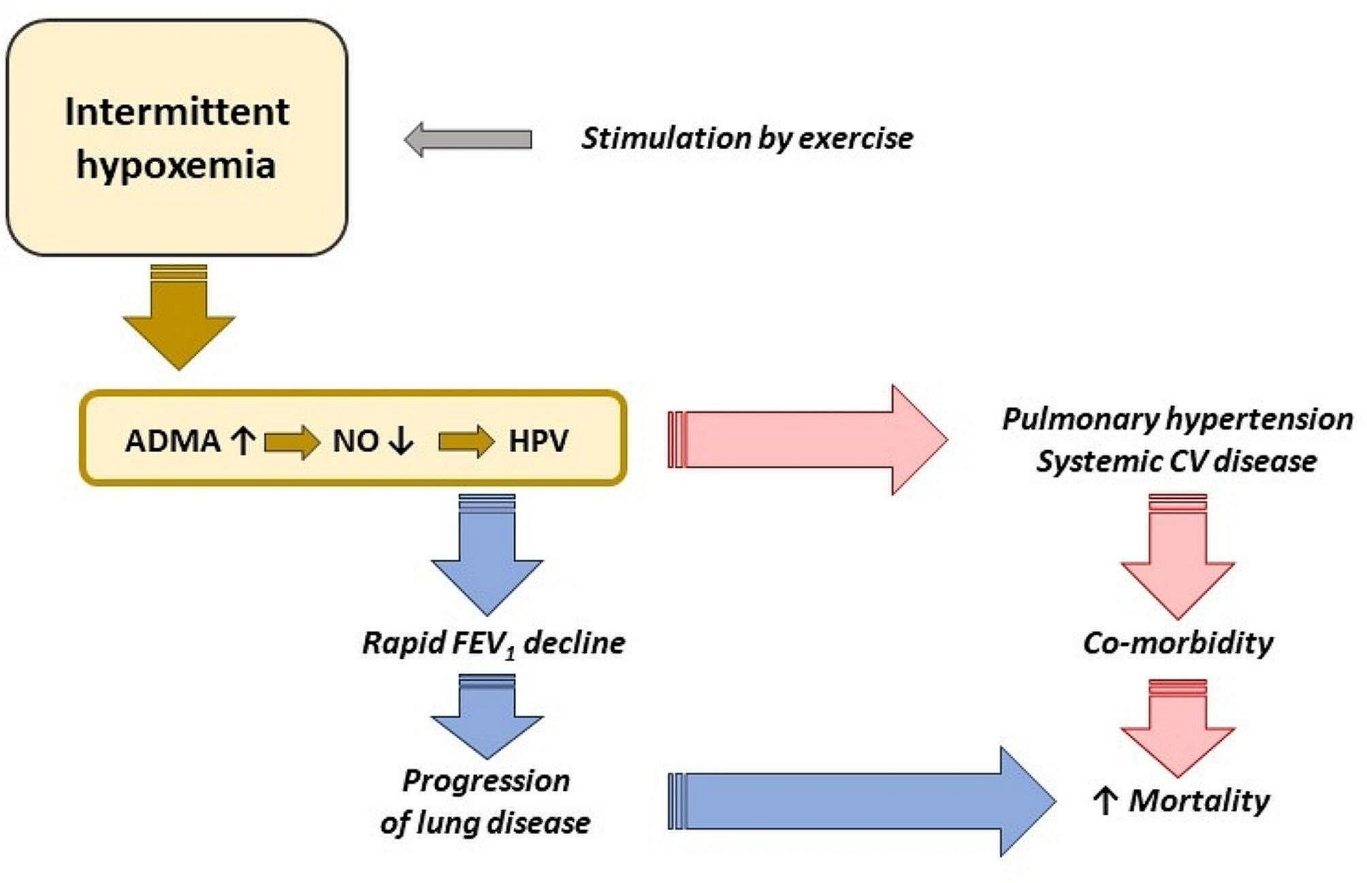
Intermittent hypoxemia, as caused by co-morbid obstructive sleep apnea, has been shown to act as a driver of rapid lung function decline, systemic cardiovascular co-morbidity, and excess mortality. Exercise is another well-known trigger of intermittent, exertional hypoxemia. Previous research has revealed that hypoxia causes dysregulation of the endothelial L-arginine – dimethylarginine – nitric oxide pathway, causing excess hypoxic pulmonary vasoconstriction. We hypothesize that dysregulation of this important pulmonary vascular signalling pathway also affects the rate of decline of lung function and, by spillover of dimethylarginines from the pulmonary into the systemic circulation, triggers systemic co-morbidities, and thereby contributes to poor prognosis

Research by our group and others has shown in recent years that the vascular L-arginine – dimethylarginine – nitric oxide pathway becomes dysregulated in hypoxia [36]. Intermittent hypoxemia caused by exercise may mimic the co-morbidity of COPD with sleep apnea syndrome, also known as overlap syndrome [19]. Patients with overlap syndrome are characterized by an extraordinarily poor prognosis as compared to patients with COPD alone [9, 37]. We have shown in the population-based BOLD study that patients with overlap syndrome are show significantly elevated plasma concentrations of ADMA [38]. ADMA is a known biomarker of mortality in pulmonary arterial hypertension [39], in cardiovascular diseases, and in the general population [40, 41]; its spillover into the systemic circulation may cause systemic cardiovascular co-morbidities such as the ones often found in COPD patients [42]. It may be one cause for systemic vascular endothelial dysfunction in association with airway inflammation in COPD patients [43]. We propose that dysregulation of this pathway in the lungs may promote the worsening of lung function and be one cause of co-morbidities that also impact on the patients’ prognosis (Fig. 2).

The PROSA study sets out to investigate, by three different means, the prevalence of exertional desaturation in COPD patients at a relatively early stage. The 15-sec breath-hold test has been described to unveil sensitivity of COPD patients to intermittent hypoxemia [20]; it is an easily feasible and versatile test in clinical practice. The six-minute walk test certainly is the most often used exercise test in chronic diseases; it has been reported to generate a reliable diagnostic yield for exertional desaturation when continuous recording of SpO_2_ is performed [19]; therefore, it was selected as the primary determinant for stratification of COPD patients with or without exertional hypoxemia in the PROSA Study. Step tests on a bicycle ergometer cause the most effort in terms of personnel and equipment; however, this procedure can be expected to generate the best reproducibility of results; ergometer step tests have been shown to allow detection of exertional desaturation with continuous SpO_2_ recording in COPD patients [21]. In PROSA, all three approaches will be compared for their diagnostic sensitivities and specificities.

Non-invasive methods for assessing cardiac function, estimating pulmonary arterial pressure, and measuring systemic endothelium-mediated vasodilation will be applied to better understand the hemodynamic and vascular functional differences between COPD patients with and without exertional desaturation. This will be accompanied by biomarker measurements in blood samples taken throughout the study’s follow-up period of three years, allowing to keep a closer eye on the underlying biochemical mechanisms.

Clinical follow-up with classical spirometric lung function testing will allow us to address the primary endpoint of the study, i.e., the difference in decline of FEV1 expressed as percent of the lower limit of normal as published in [26] during follow-up between patients with and without exertional hypoxemia, respectively. This is a robust endpoint that is based on a ubiquitously available clinical method, ensuring that our results will be easily transferable into routine pneumological patient care. Beyond this, we will also analyze total mortality during follow-up and hospitalization rates for COPD exacerbation and for other causes as secondary clinical endpoints.

**Fig. 3:**
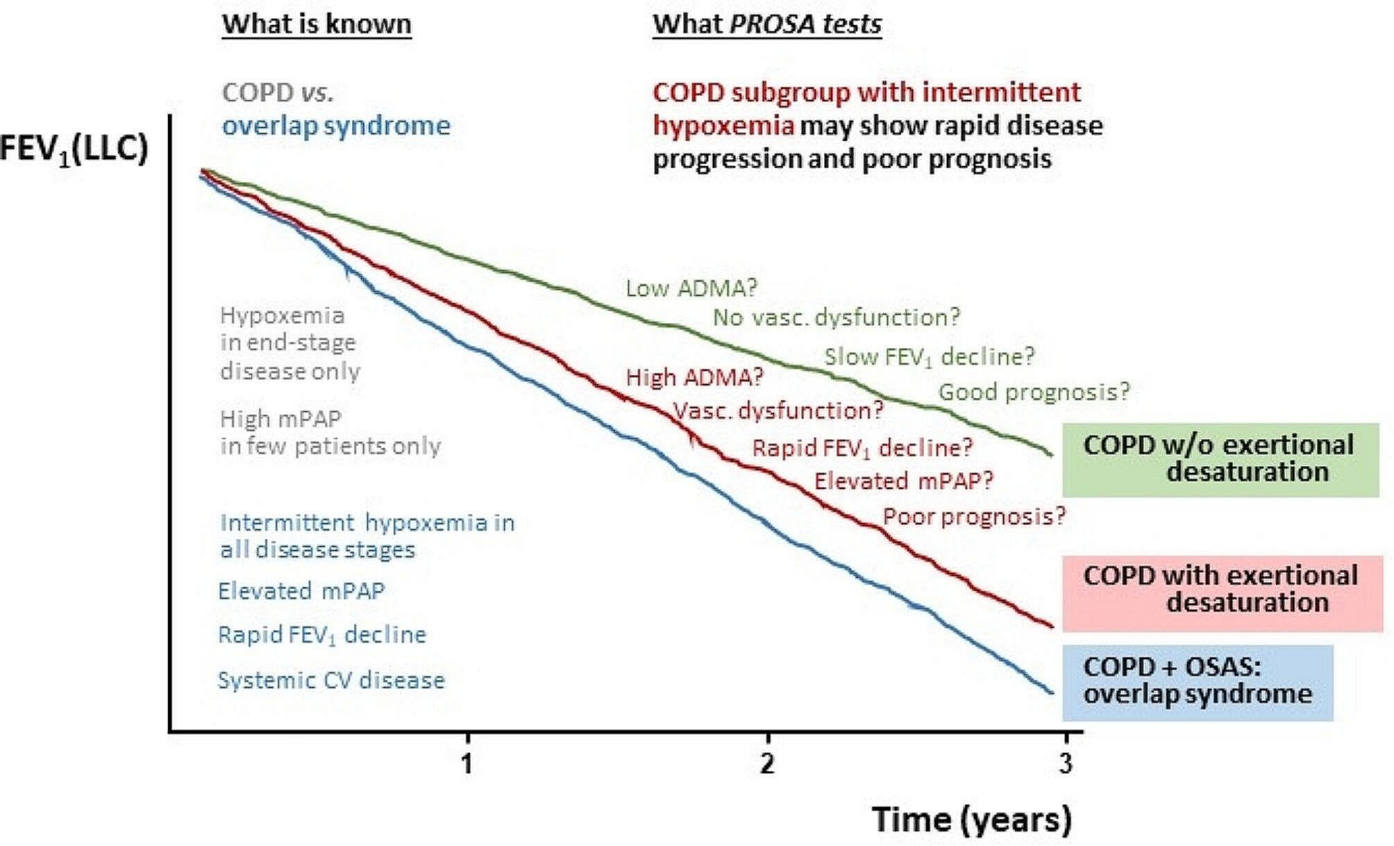
Currently hypoxemia is accepted as a clinical feature in the end-stage of some patients’ disease course, requiring long-term oxygen therapy. By contrast, it is well known that patients with overlap syndrome, i.e., concurrent COPD and obstructive sleep apnea, develop a more rapid decline in lung function, elevated pulmonary artery pressure, and systemic co-morbidity. The PROSA Study was designed to test the hypothesis that COPD patients who experience intermittent, exertional hypoxemia also have a more rapid decline in lung function than those without exertional desaturation. If this hypothesis will be proven, exercise testing may be introduced as a prognostic functional test in early-stage COPD. PROSA will also include biomarker analyses and additional functional studies to highlight potential signalling pathways that may link exertional hypoxemia to lung function decline, pulmonary artery pressure, and systemic cardiovascular dysfunction, and thereby aim to shed light on possible pathomechanisms and potential novel targets for therapy

Aside from this, we will strive to better understand the pathophysiology linking exertional hypoxemia with progression of lung disease. We have evidence supporting a role for the pulmonary L-arginine – ADMA – NO pathway in modulating the response of the lung to intermittent hypoxia, and we will test in PROSA whether L-arginine-related metabolites may be suitable as biomarkers for differentiating COPD patients with and without exertional hypoxemia and/or COPD patients with good or poor prognosis, respectively. Biomarkers have become important clinical diagnostic tools that also help in therapeutic decision making. Thus, if the biomarkers we will analyze show predictive value for differentiating COPD patient subgroups, this may aid in risk assessment in clinical routine as well. Also, therapeutic intervention in this pathway has been tested by various means in other diseases; it may help to find more personalized treatments options for the subgroup of COPD patients with exertional hypoxemia and thus stimulate future therapeutic trials. The overall hypothesis for PROSA is depicted schematically in Fig. 3.

## Data Availability

No datasets were generated or analysed during the current study.

## Abbreviations

COPD: Chronic obstructive pulmonary disease
FEV1: Forced expiratory volume in 1 s
FEV1(LLN): Forced expiratory volume in 1 s, expressed as percent of the lower limit of normal
FVC: Functional vital capacity
GOLD: Global Initiative for obstructive lung disease
NO: Nitric oxide
OSAS: Obstructive sleep apnea
PROSA: Prospective observational study to assess the influence of intermittent hypoxemia on lung function decline, morbidity, and mortality in COPD patients
6MWT: Six-minute walk test
SpO_2_: Oxyhemoglobin saturation measured by pulse oximetry
